# Translation, adaptation, and clinical validation of the Premature Ejaculation Diagnostic Tool in Spanish (Colombia)

**DOI:** 10.1093/sexmed/qfac017

**Published:** 2023-01-30

**Authors:** Pablo Vallejo-Medina, José Pablo Saffon, Ana Álvarez-Muelas

**Affiliations:** SexLabKL, School of Psychology, Fundación Universitaria Konrad Lorenz, Bogotá, Colombia; Boston Medical Group, Colombia; Centro de Investigación Mente, Cerebro y Comportamiento, Universidad de Granada, 18011, Granada, Spain

**Keywords:** PEDT, premature ejaculation, Colombia, Spanish, *ICD-10*, sexual function, men

## Abstract

**Background:**

Premature ejaculation is the most prevalent form of sexual dysfunction in men. The Premature Ejaculation Diagnostic Tool (PEDT) is an instrument used to evaluate premature ejaculation. It offers adequate psychometric properties and good reliability.

**Aim:**

To adapt and validate a Colombian version of the PEDT in Colombian clinical and nonclinical samples.

**Methods:**

Two samples were used in this study. The first was made up of 1110 men who were recruited to evaluate validity and reliability. Their ages ranged from 19 to 65 years (mean ± SD, 39.71 ± 12.53). The second sample included 123 men (66.7%) who did not meet diagnostic criteria for premature ejaculation per the *International Statistical Classification of Diseases and Related Health Problems* (*ICD-10*), while 33.3% met *ICD-10* criteria for this dysfunction. Their ages ranged from 18 to 65 years (34.19 ± 12.65). Scores were used to calculate the cutoff.

**Outcomes:**

A translated and adapted version of the PEDT was developed specifically for Colombia. All participants completed the Colombian version of the PEDT, a sociodemographic questionnaire, the Colombian version of the Massachusetts General Hospital–Sexual Functioning Questionnaire, and a semistructured interview based on the *ICD-10*.

**Results:**

The results showed adequate psychometric properties and satisfactory internal consistency and confirmed the 1-dimensional factorization of the scale. According to *ICD-10* criteria, the study also confirmed significant differences between participants who self-reported premature ejaculation and those who did not. In addition, it showed adequate evidence of convergent validity, with a moderate correlation with sexual functioning scores. As a result, the cutoff point was set to 10.5, with an area under the curve of 96.8%. Therefore, a score ≥11 points suggested the presence of premature ejaculation.

**Clinical Translation:**

The current Colombian version of the PEDT is a useful instrument that determines the presence of premature ejaculation that is compatible with *ICD-10* criteria.

**Strengths and Limitations:**

The Colombian version of the PEDT presents evidence of reliability and validity, a confirmed 1-dimensional factorization, and a cutoff point for Hispanic populations. More in-depth evaluation of the diagnosis of premature ejaculation is required, and further research among other Spanish-speaking countries and sexual minorities is recommended.

**Conclusion:**

The Colombian version of the PEDT is a psychometric adequacy tool for evaluating and diagnosing premature ejaculation, following the *ICD-10* criteria.

## Introduction

Male sexual dysfunction affects around 31% of the population, and its incidence increases with age.^1-3^ It has been observed to have a negative effect on general well-being^4^ and has been associated with low self-esteem,^5^ mental health problems,^6^^,^^7^ intimacy issues, and decreased marital functioning and sexual satisfaction.^8^ Premature ejaculation (PE) is one of the most common types of sexual dysfunction (5%-35%).^9-12^ However, the definition proposed by the International Society for Sexual Medicine^13^ and the diagnostic criteria of the *Diagnostic and Statistical Manual of Mental Disorders* (DSM-5)^14^ point to a much lower incidence (approximately 4%).^13^

The person’s medical and sexual history is taken into account when evaluating PE.^15^ Yet, it is important to consider criteria such as intravaginal ejaculatory latency time, ejaculation control, and distress.^16^ The Premature Ejaculation Diagnostic Tool (PEDT)^17^^,^^18^ is an instrument to evaluate PE.^19^ This self-report tool consists of 5 items grouped into a single factor. The items refer to criteria of the *DSM-IV-TR* (ie, text revision)^20^: ejaculation control, frequency of PE, minimum sexual stimulation, anxiety, and interpersonal difficulty. The scale obtained adequate psychometric properties and good evidence of reliability, with a test score of 0.73 and Cronbach alpha score of 0.71. The sensitivity and specificity of the scale were also analyzed, and the scale’s cutoff point was obtained: scores ≤8 indicated the absence of PE; 9 and 10, the probable presence of PE; and ≥11, the presence of PE.^17^

Although evidence of the reliability and validity of the PEDT has been reported, the instrument still needs to be adapted to different populations.^19^ Thus, the scale has been translated into other languages (English, Finnish, French, German, Hebrew, Hungarian, Italian, Norwegian, Polish, Portuguese, Spanish, Swedish, and Turkish),^18^ but it has been adapted and validated only in Turkey,^21^ Korea,^19^ Finland,^22^ Iran,^23^^,^^24^ and China.^25^^,^^26^

The wide range of reference figures on the prevalence of PE highlights the need to correctly evaluate the dysfunction. The PEDT has not been adapted or validated for Spanish-speaking populations, likely because cultural differences and linguistic expressions need to be taken into account.^27^ For this purpose, the present study adapted and validated the PEDT for Colombian adults. The research question was to find evidence of the reliability and validity of the Colombian version of the PEDT.

## Methods

### Participants

Two independent samples were used for this study. The first, consisting of 1110 men, was used to perform a classic validation of the scale. The general population and a group of outpatients from a Colombian sexology clinic (Boston Medical Group) participated in this study. The non-PE group included men who did not report PE (n = 541), whereas outpatients who self-reported PE issues (n = 569) composed the PE group. The participants’ ages ranged from 19 to 65 years (mean ± SD, 39.71 ± 12.53). Of the total sample, 80.2% reported being exclusively heterosexual, 5.8% exclusively homosexual, 2.4% asexual, and 10.4% indicated different levels of bisexuality. All men in these samples were Colombian, and their distribution by place of residence showed that 39.1% lived in Bogotá, 9.9% in Medellín, 6.1% in Cali, 3.6% in Barranquilla, and 39.8% in other Colombian cities. Table 1 provides a description of the variables for this sample. The inclusion criteria were being aged ≥18 years, having signed an informed consent form, living in Colombia, and being literate.

**Table 1:** Demographic information: sample 1.^a^

	**PE (n = 569)**	**Non-PE (n = 541)**	**Contrast**
Age, y	39.30 ± 12.84	40.10 ± 12.23	*t*(1108) = −1.06, *P =* .06
Sexual orientation			χ^2^(7) = 53.41, *P* < .001
Asexual	4	0.9	
Heterosexual	86.30	76.20	
Bisexual	7.60	13.30	
Homosexual	2.30	9.40	
Couple relationship			χ^2^(1) = 13.21, *P* < .001
Yes	78	68	
No	22	32	
Marital status			χ^2^(5) = 30.59, *P* < .001
Married	37	26	
Single	29	45	
Separated	10	10	
Widowed	0.7	0.6	
Common-law marriage	21	18	
Medication			
Antihypertensive	6	4.1	χ^2^(1) = 2.11, *P* = .146
Antidepressant	3.2	2.4	χ^2^(1) = 0.60, *P* = .442
Antipsychotic	0.5	0.2	χ^2^(1) = 0.91, *P* = .341
Anxiolytic	1.1	2.2	χ^2^(1) = 2.35, *P* = .125
Somnifer	1.2	2	χ^2^(1) = 1.12, *P* = .290
Disease			χ^2^(16) = 694.05, *P* < .01
Apoplexy	0	0.2	
High/low blood pressure	14.80	11.80	
Thyroid problems	0	4.80	
Heart problems	1.80	2.60	
Cerebral infarction	0.4	0.6	
Urologic problems	1.60	8.30	
Psychiatric diagnosis	0	2.2	
Anxiety	0	25.90	
Depression	0	6.3	
Alcohol abuse	0	7	
Drug abuse	1.20	0.7	
Diabetes	0	10	
Cancer	7	0	
Neurologic problems	0.2	2	
Blood-related problems	3.2	0	
Sexually transmitted infections	0	7.2	

Abbreviation: PE, premature ejaculation.

a Values are presented as mean ± SD or %.

The second sample consisted of 123 men and was used to examine the cutoff point for this version of the PEDT, which requires a subsample classified according to diagnostic criteria. Two-thirds of these men (66.7%, n = 82) were volunteers who did not meet the *ICD-10* diagnostic criteria for PE.^28^ This group was labeled *non-PE*. The remaining participants (33.3%, n = 41) were men who met the *ICD-10* diagnostic criteria for PE. The age range of the participants was 18 to 65 years (34.19 ± 12.65). More information on both groups can be found in Table 2. The inclusion criteria for this sample were the same as those in the first sampling procedure, except that the PE group was required to meet the *ICD-10* diagnostic criteria for premature ejaculation.^28^

**Table 2:** Demographic information: sample 2.

	**PE (n = 41)**	**Non-PE (n = 82)**	**Contrast**
Age, y	35.76 ± 10.40	33.40 ± 13.63	*t*(121) = 0.97, *P* < .01, *d* = 0.19
Sexual orientation			χ^2^(5) = 7.36, *P* = .19
Asexual	0	3.70	
Heterosexual	97	75.60	
Bisexual	0	7.20	
Homosexual	3	13.40	
Couple relationship			χ^2^(1) = 0.79, *P* = .37
Yes	58.50	50	
No	41.50	50	
Marital status			χ^2^(3) = 7.07, *P* = .70
Married	36.90	26.20	
Single	29.50	44.70	
Separated	10.50	10.40	
Widowed	0.7	0.6	
Common law marriage	21.10	17.60	
Medication			χ^2^(1) = 2.10, *P* = .14
Does use	4.90	13.40	
Does not use	95.10	86.60	
Disease			χ^2^(1) = 0.01, *P* = .89
Yes	41.50	35.40	
No	58.50	64.60	

Abbreviation: PE, premature ejaculation.

a Values are presented as mean ± SD or %.

### Instruments

#### Premature Ejaculation Diagnostic Tool

The PEDT is a self-reporting instrument that consists of 5 items that evaluate the presence or absence of premature ejaculation.^17^^,^^18^ The items correspond to the following areas: ejaculation control (items 1 and 3), frequency of PE (item 2), minimum sexual stimulation (item 3), anxiety (item 4), and interpersonal difficulty (item 5). Response options were given on a Likert-type scale, with possible scores between 0 and 4. Higher values indicate more PE symptoms. The reliability of the instrument was reported as adequate in the original study,^17^ with a test-retest reliability of 0.73 and Cronbach alpha of 0.71.

#### Massachusetts General Hospital—Sexual Functioning Questionnaire

The present study used the Colombia-validated male version of the Massachusetts General Hospital–Sexual Functioning Questionnaire (MGH-SFQ), composed of 5 items: sexual desire, sexual arousal, orgasm, erection, and general satisfaction.^29^^,^^30^ The questionnaire uses a 5-point Likert-type scale (0-4), where scores <2 indicate possible sexual problems. In the present study, the Cronbach alpha was 0.91.

#### Diagnostic interview

A semistructured interview based on the *International Statistical Classification of Diseases and Related Health Problems* (*ICD-10*)^28^ was used to evaluate the occurrence of PE. Five questions were asked to confirm or reject the presence of PE following the diagnostic criteria of the World Health Organization^28^^,^^31^:

(1) In the last six months, have you been able to engage in sexual intercourse as you wish?
(2) In the last six months, how often did you ejaculate before you wished?
(3) In the last six months, does ejaculation occur before the beginning of sexual intercourse or within 15 seconds of the beginning of intercourse?
(4) In the last six months, has ejaculation occurred in the absence of sufficient erection to make intercourse possible?
(5) In the last six months, how often have you engaged in sexual activity (masturbation, oral sex, or penetration)?

#### Sociodemographic questionnaire

The sociodemographic questionnaire was composed of various items that collected information on age, sex, educational level, sexual orientation, couple relationship, reports on psychological and medical diagnoses, and drug use, among other areas.

### Procedure

The study procedure was based on the 2 Spanish-language versions of the PEDT (the US and Spain versions) available at https://www.pfizerpcoa.com/. Neither appeared to be easy for Colombians to understand, and neither has been validated for this population. Therefore, we decided to translate and adapt a new version for the South American country of Colombia. For this purpose, 2 independent translators conducted 2 separate certified translations. These translations were discussed by a group of translators, psychometry experts, and sexologists. After the discussion, a new version was created by using the contributions of both translators. This new version was back-translated into English and analyzed for differences in content. This process was conducted according to international guidelines.^32-35^ The final version is available at https://www.pfizerpcoa.com/ and is included in the supplementary material.

The present study used 2 types of samples, and a portion of the participants was recruited via an agreement established with the Boston Medical Group clinic between August 2018 and August 2019. The first sample included men who responded to a web-based survey on SurveyMonkey that was distributed through social networks (Facebook and Twitter) and the clinic’s updated directory. The second sample included men who responded to a pencil-and-paper survey and reported PE during their first visit to the clinic. Participants without PE were evaluated in libraries, universities, study halls, and training rooms. Thus, all samples were incidental and nonprobabilistic. Individual evaluations lasted approximately 10 minutes.

### Ethical statement

This study is derived from a research project (No. 55270151) revised and approved by an ethics committee of the Fundación Universitaria Konrad Lorenz. All subjects signed an informed consent agreement, and confidentiality was maintained throughout the study. Openly accessible data were not considered in these consent agreements. Participation was voluntary and anonymous.

### Data analysis

The data were analyzed with RStudio.^36^ Given the polytomous nature of the PEDT response scale, all results derived from the matrix were obtained from a polychoric matrix. For instance, the alpha presented is not the Cronbach alpha but ordinal alpha. A structural equation model was created with the *lavaan* software package.^37^ Given the lack of compliance with multivariate normality, the robust estimation method chosen was diagonally weighted least squares, mean and variance adjusted. The indices of fit and their thresholds were as follows: comparative fit index ≥0.95, Tukey-Lewis index ≥0.95, and standardized root mean residual ≤0.06. The root mean square error of approximation, a widely employed estimator, was discarded because of the bias observed in models with few degrees of freedom.^38^ Receiver operating characteristic (ROC) curves were generated via the *pROC* package,^39^ and the same software was used to obtain 95% CIs with 2000 stratified bootstrap replicates. Finally, we used *ggplot2* to create some graphics^40^ and Psych to conduct psychometric analyses.^41^

## Results

### Psychometric properties of the items

The results associated with the psychometric properties of the items (Table 3) showed an adequate response distribution, with means close to the theoretical mean of the scale (2 points) and deviations >1. Both distributions drift from the Mardia test for multivariate normality (*P* < .01 for skewness and kurtosis). Additionally, high corrected item-total correlations (always >0.60) and adequate reliability were observed, while eliminating the items failed to increase the correlation.

**Table 3:** Selected psychometric properties of items.

**PEDT**	**Mean ± SD**	**Skewness**	**Kurtosis**	**c**_**i-t**_^**c**^	**α item** ^**a**^
Item					
1	1.48 ± 1.29	0.41	−1.02	0.65	0.90
2	1.87 ± 1.49	0.16	−1.42	0.85	0.86
3	1.30 ± 1.36	0.65	−0.88	0.71	0.89
4	1.68 ± 1.42	0.27	−1.34	0.86	0.86
5	2.39 ± 1.27	−0.52	−0.86	0.70	0.89
Total	8.71 ± 5.57				0.90

Note: α -item = ordinal alpha if item is deleted; c_it_^c^ = corrected item-total polychoric correlation; *M* = mean; *SD* = Standard Deviation; α = ordinal alpha.

a Ordinal alpha if item is deleted. The final value in the column indicates the total ordinal alpha.

### Confirmatory factor analysis

After obtaining optimal results for the properties of the scale items, we tested its unidimensionality, which has already been carried out for other cultures and countries. We confirmed the 1-dimensional model directly, omitting error covariation. Table 4 shows the standardized weights for the items, with errors and explained variance. The model’s fit indices were adequate (χ^2^ = 63.94, *df* = 5, *P* < .01, standardized root mean residual = 0.036, comparative fit index = 0.99, Tukey-Lewis index = 0.99).

**Table 4:** One-dimensional model: standardized weights, standard errors, and explained variance. ^a^

**PEDT**	**Standardized weight, λ**	**SE**	**Explained variance, *R*** ^**2**^
Item			
1	0.69	0.017	0.47
2	0.90	0.009	0.81
3	0.77	0.015	0.60
4	0.91	0.008	0.84
5	0.77	0.014	0.60

Abbreviation: PEDT, Premature Ejaculation Diagnostic Tool.

a χ^2^ = 63.94, *df* = 5, *P* < .01, standardized root mean residual = 0.036, comparative fit index = 0.99, Tukey-Lewis index = 0.99.

### Discriminant and convergent validity

**Figure 1:**
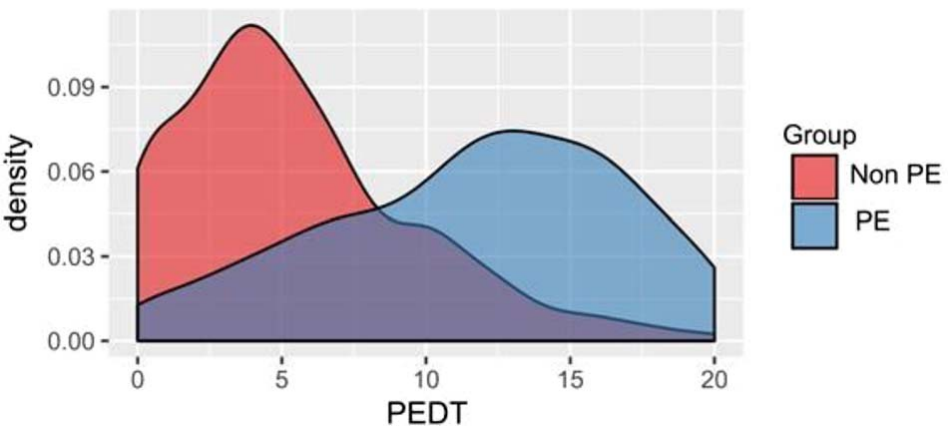
Density plot for groups reporting the absence and presence of premature ejaculation (PE). Mean ± SD: Non-PE, 5.52 ± 4.14; PE, 11.54 ± 5.12; *t*(1107) = 21.46, *P* < 0.01, *d* = 1.29.

Discriminant validity was tested when the unidimensionality of the scale was confirmed. For this purpose, we compared the scores of men who did not self-report PE with men who self-reported PE and had visited a clinic seeking help with their PE problems. Significant and pronounced differences between the groups are shown in Figure 1. Convergent validity showed a moderate correlation (*r* = -.31^**^) between the PEDT and MGH-SFQ.

### ROC curves

**Figure 2:**
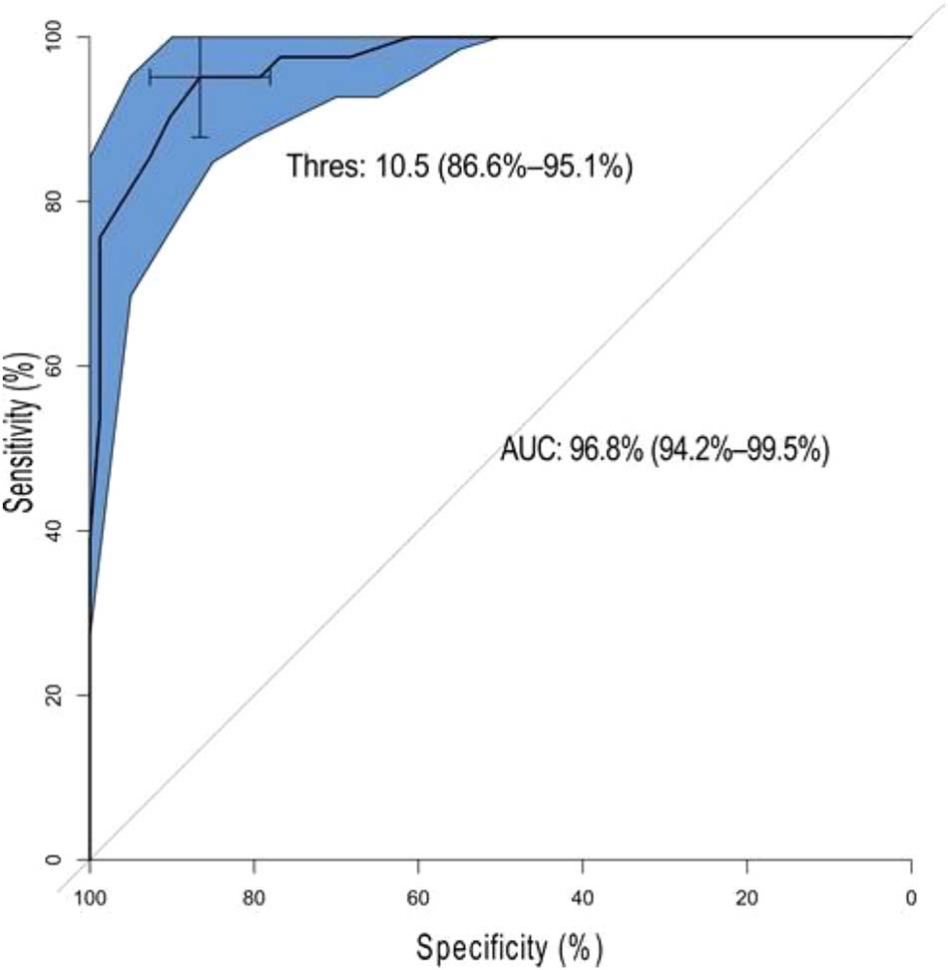
Receiver operating characteristic curve plot. AUC, area under the curve (95% CI); Thres, threshold (specificity, 88.6%; sensitivity, 95.1%). The complete 95% CI is shown in blue (gray if in print version).

The cutoff point of the Colombian version of the PEDT was evaluated. The ROC curve procedure was performed for this purpose. The results showed that the cutoff point with the highest balance between sensitivity and specificity was 10.5. The area under the curve for this value was 96.8%. Figure 2 shows the additional information.

## Discussion

PE has a consequential impact on the quality of life of patients and their partners.^40^ PEDT has proven to be a useful evaluation tool for identifying PE.^43^ This study, which represents the first instance of PEDT validation for the Spanish language, confirmed that the items in the adapted questionnaire have adequate psychometric properties. It also demonstrated the 1-dimensional structure of the instrument; its adequate reliability, convergent, and discriminant capacities; and a cutoff point similar to that found in other studies.

First, evidence of the psychometric properties of the questionnaire was found. The corrected item-total correlation indices presented adequate values, as in the original study.^17^ The ordinal alpha value was 0.90, which is higher than the originally reported value^17^^,^^18^ of 0.70 and the values found in Turkey (0.77)^21^ and China (0.77 and 0.79).^25^^,^^26^ However, these differences are not noteworthy, and the values reported by other authors (eg, Iran,^24^ 0.89; Finland,^22^ 0.89 and 0.88; South Korea,^19^ 0.93) are similar to those observed in the present study. In any case, these measures of reliability suggest that the scale can be used for clinical and research purposes. It was also observed that the reliability of the scale remained unchanged when any of the items were eliminated.

The unidimensionality of the Colombian adaptation of the scale was confirmed, as was the case with the original version^17^^,^^18^ and the Iranian^24^ and Turkish^21^ adaptations. Standardized weight scores ranged between 0.69 and 0.90, which are adequate values and generally higher than those found by Symonds et al,^17^ which range from 0.41 to 0.88.

Significant differences were observed between the scores of the participants who self-reported PE and those who did not (large effect size). Moreover, the moderate correlations observed with the MGH-SFQ indicated adequate criterion validity. Correlations were not higher because the MGH-SFQ is a general sexual performance scale, not specifically intended to assess PE.

Moreover, our analyses showed that a score ≥11 points indicates the possible presence of PE. Despite the significant differences by age in this sample, the effect size was small. Therefore, its effect on the results was minimal. Moreover, this cutoff score is the same as that reported by the original authors^17^ (11 points) and is similar to that cited by Tang et al,^16^ Kam et al,^19^ and Jiang et al.^26^ Yet, this value is much different from that obtained by the Finnish adaptation,^22^ whose cutoff point was set at 17 points. This could be due to the fact that in the Finnish scale, response options range from 1 to 5 points, whereas in the original version, they range from 0 to 4. Thus, a simple correction (17 – 5 = 12) allows for a certain consensus regarding the cutoff point of the scale. We should also highlight that the original PEDT used the *DSM-IV-TR* criteria,^20^ but it appears to be sensitive to the *ICD-10* diagnostic criteria.^28^

Among the limitations of this study, the use of the instrument is limited to the evaluation process because it allows for the detection of PE. Although the current version of the PEDT seems to be sensitive to *ICD-10* diagnostic criteria, we recommend a more in-depth evaluation of the diagnosis of PE. In addition, the adaptation and validation of the scale were performed in the Colombian population, which is mostly heterosexual. For this reason, we suggest further research on its use with other Spanish-speaking populations and sexual minorities. Moreover, future research could examine evidence reliability with test-retest analysis.

## Conclusions

In conclusion, this study presents evidence for the reliability and validity of the Colombian version of the PEDT. This version is a psychometric adequacy tool for evaluating and diagnosing PE following *ICD-10* criteria in clinical and research environments. Therefore, it is a useful evaluation tool for identifying PE in Colombia.

## Funding

This study was made possible thanks to funding provided by the Fundación Universitaria Konrad Lorenz via research project 55270151, granted to the corresponding author.

*Conflicts of interest*: None declared.
