# Correction to: Translation, adaptation, and clinical validation of the Premature Ejaculation Diagnostic Tool in Spanish (Colombia)

**DOI:** 10.1093/sexmed/qfae032

**Published:** 2024-05-17

This is a correction to: Pablo Vallejo-Medina, Pablo José Safón, Ana Álvarez-Muelas, Translation, adaptation, and clinical validation of the Premature Ejaculation Diagnostic Tool in Spanish (Colombia), *Sexual Medicine*, Volume 11, Issue 1, February 2023, qfac017, https://doi.org/10.1093/sexmed/qfac017.

In the originally published version of this manuscript, author José Pablo Saffon's name erroneously appeared as Pablo José Safón.

This error has been corrected.

